# Erythroferrone antagonism of BMPs is governed by a composite heparin-binding motif

**DOI:** 10.1016/j.jbc.2026.113250

**Published:** 2026-06-12

**Authors:** Eleanor M. Mast, Lucija Hok, Edmund A.E. Leach, Thomas B. Thompson

**Affiliations:** Department of Molecular and Cellular Biosciences, University of Cincinnati, Cincinnati, Ohio, USA

**Keywords:** iron, bone morphogenetic protein (BMP), analytical ultracentrifugation, molecular dynamics, heparin-binding proteins, heparan sulfate, mutagenesis, protein purification, flow cytometry, bio-layer interferometry (BLI), erythroferrone, transforming growth factor beta family (TGF-β), C1Q/TNF-related protein family (CTRP)

## Abstract

Iron homeostasis is regulated by bone morphogenetic protein (BMP) signaling, which induces hepcidin to negatively regulate serum iron levels and iron import. After a major blood loss event, developing erythroblasts produce erythroferrone (ERFE), which inhibits hepatocyte BMP signaling to increase serum iron levels and drive their maturation to erythrocytes. While ERFE was recently shown to contain a heparin-binding motif (HBM), its mechanistic significance remains poorly understood. Here, we establish that the ERFE HBM is essential for BMP inhibition and uncover a novel ternary mechanism for ligand antagonism. Using biophysical assays and molecular dynamics (MD) simulations, we show that heparin/heparan sulfate (HS) simultaneously engages with the HBM of both ERFE and BMP6 to stabilize a high-order inhibitory complex. This complex exerts far greater affinity for HS in the extracellular matrix than ERFE alone, supporting potent, ligand-dependent localization to the cell surface. Importantly, we demonstrate that ERFE preferentially engages the BMP6:HS complex over other BMP ligands, suggesting modes of ligand-HS interactions are key determinants for selectivity. Together, these findings reframe ERFE as a matrix-assisted antagonist that exploits HS as a structural co-factor for BMP antagonism and gives insight into the mechanism for tissue-restricted BMP antagonism of ERFE.

Erythroferrone, or ERFE, is the erythroid regulator of hepcidin and gut iron import and its misregulation can cause iron overload (1, 2, 3, 4). Under basal conditions, high serum iron levels upregulate liver endothelial cell production of bone morphogenetic proteins (BMPs) (5, 6). BMPs induce hepcidin expression in hepatocytes, which negatively regulates iron import to the bloodstream (7, 8, 9, 10). This negative regulatory pathway is essential for preventing iron overload, as the body has no process for removal of iron from the body after it is imported to the bloodstream. However, the body must occasionally turn off this pathway to allow for increased serum iron levels (11). Following a blood loss event, developing erythroblasts secrete ERFE. Elevated ERFE inhibits the negative regulation of iron by inhibiting BMP-induced hepcidin synthesis. This increases serum iron levels and supports the rapid generation of hemoglobin and the subsequent maturation of erythroblasts to erythrocytes (1). However, pathogenic levels of ERFE contribute to iron overload in mouse models of β-thalassemia, where constant hypoxia leads to constitutive erythropoiesis (2, 3, 4). Thus, continual ERFE secretion turns off the natural regulatory system, leading to iron overload. Due to ERFE’s role in disease and iron homeostasis, a greater understanding of its unique molecular mechanism of action may enable the development of novel therapeutics and further our understanding of BMP inhibitors.

ERFE is a member of the C1Q/TNF-related protein (CTRP) superfamily and shares the family’s distinctive structure: a 100 to 200 amino acid N-terminal unstructured domain and a C-terminal trimerizing C1Q/TNF-like globular domain (12). The N-terminal region contributes to oligomerization and contains a proline-rich, trimerizing GXY collagen-like repeat (CLR) as well as cysteine residues that form interchain disulfide bonds (13). Notably, ERFE diverges from most other family members by possessing an unusually short CLR and a distinct cysteine arrangement. Within the family, ERFE is the only member known to inhibit BMP ligands (13). While ERFE can form higher order species, the basic unit for BMP inhibition is a trimer (14). ERFE binds BMP ligands directly through a conserved α-helix within the unstructured domain termed the ligand binding domain (LBD), which is positioned N-terminally from the CLR and cysteines (Fig. 1*A*) (14).

**Figure 1:**
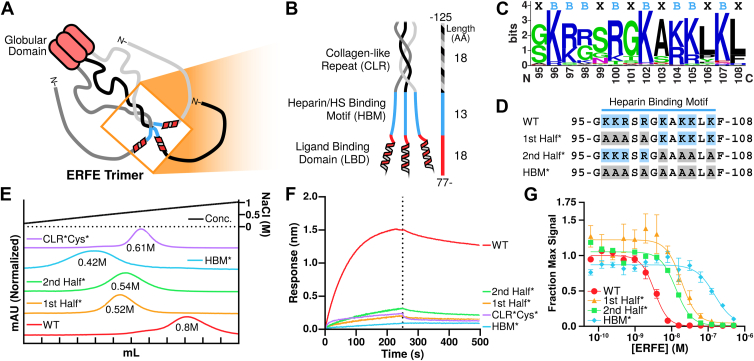
**ERFE contains a conserved heparin binding domain critical for heparin affinity and activity.***A and B*, schematic of the ERFE trimer highlighting important regions of the unstructured domain located between residues 77 and 125. *C*, WebLogo representation of ERFE HBM conservation across mammals. *D*, sequence alignment of the WT human ERFE HBM and HBM mutants created. *E*, heparin affinity chromatography analysis of ERFE mutants, eluted at a 0–1 M NaCl gradient. The salt concentration in molar is listed under the midpoint of each peak. *F*, BLI analysis of 240 nM WT and mutant ERFE binding to SA-Biotin-Heparin tips. Curves are a representative sample of n = 4 independent experiments. *G*, WT and mutant ERFE were titrated against 2.5 nM BMP6 in a cell-based luciferase reporter assay. Representative curve of n = 3 biological replicates is shown. IC_50_ values, ±SEM: WT (4.16 ± 0.45 nM), first half∗ (17.7 ± 0.46 nM), second half∗ (11.0 ± 1.10 nM), HBM∗ (133 ± 9.13 nM).

BMPs constitute the largest and most evolutionarily ancient subfamily of the transforming growth factor-β (TGF-β) superfamily of signaling ligands and regulate diverse processes, including embryonic patterning, bone formation, heart development, neurogenesis, and tissue homeostasis in the adult (15, 16, 17, 18). Like other family members, BMPs function as disulfide-linked dimers that engage two type I receptors and two type II receptors, triggering SMAD-dependent changes in DNA transcription (16, 18). BMPs can be broadly categorized into 4 main subclasses based on sequence conservation: class I (BMP2/4), class II (BMP5/6/7/8a/8b), class III (GDF5/6/7), and class IV (BMP9/10). ERFE is the only BMP antagonist to primarily inhibit class II BMPs and additionally inhibits the BMP2/6 heterodimer, the ligand proposed to drive hepcidin induction *in vivo* (3, 19).

BMPs signaling is further regulated in a subclass-specific manner by heparin and heparan sulfate (HS) proteoglycans (HSPGs). Heparin is a heavily sulfated polysaccharide (3–30 kDa; mean ∼15 kDa) commonly used clinically as an anticoagulant, but it does not regulate BMP signaling in its natural role (20, 21). In contrast, HS is a polysaccharide composed of heavily sulfated regions interspersed with unsulfated regions (22, 23). HS chains decorate HSPGs in the extracellular matrix (ECM) on the surface of most cells, including transmembrane HSPGs like syndecans and glypicans, along with large HSPGs in basement membranes like perlecan and agrin (22, 23). Class I BMPs are negatively regulated by HSPGs, which sequester them to the cell surface and can mediate their rapid endocytosis and degradation (24, 25). Meanwhile, Class II BMPs require HSPG interactions to induce a signal, despite exhibiting much lower affinity for HSPGs (25, 26, 27). Finally, class III and class IV BMPs have not been shown to interact appreciably with HSPGs (16, 18, 28).

Several extracellular BMP antagonists, including noggin and gremlin1/2, are regulated through interactions with the ECM (29, 30, 31). High-affinity antagonist:ECM interactions limit diffusion of the antagonist, causing it to act locally (22, 29, 31). Often, the terminal antagonist:ligand complex has higher heparin/HS affinity than the antagonist itself, which facilitates endocytosis and degradation (31, 32). Recent studies suggest ERFE can bind heparin through a positively charged patch of amino acids hypothesized to be a heparin binding motif (HBM) (33). Unlike other antagonists, however, these charged residues are suggested to be essential for BMP inhibition (33). Moreover, because ERFE functions as a circulating endocrine factor, it remains unclear how HS affinity influences its systemic activity and tissue targeting. This suggests that our current mechanism of ERFE:BMP interactions may be incomplete.

In this study, we define the molecular basis of ERFE binding to heparin/HS and establish its functional role for BMP inhibition. We demonstrate that heparin/HS stabilizes the ERFE:BMP6 complex by simultaneously engaging the heparin-binding motifs of both proteins. We also demonstrate that in the absence of BMPs, ERFE exhibits weak association with the cell surface; however, upon binding a BMP ligand, ERFE forms high-order complexes with enhanced affinity for heparin and the cell surface. Collectively, these findings support a model in which HS plays an active role in the formation of the inhibitory complex, revealing a previously unrecognized mechanism of BMP inhibition.

## Results

### ERFE contains a heparin-binding motif critical for activity

Previous studies suggested that ERFE contains a positively charged region that may mediate heparin binding and regulate ERFE activity (33). This 13-amino acid segment is located between the collagen-like repeat (CLR) and the highly conserved ligand-binding domain (LBD) (Fig. 1, *A* and *B*) (14). Comparative analysis across mammals reveals strong conservation of this region, with a high prevalence of lysine and arginine residues, interspersed with flexible, polar residues at regular intervals (Fig. 1*C*) (34). The segment has a consensus sequence of XBBBXBXBXBBXBX (B = basic residue; X = noncharged, nonaromatic residue) and is consistent with multiple canonical heparin-binding consensus sequences (35, 36).

To validate the heparin-binding motif (HBM), we generated mutants where the positive residues were replaced by alanine and assessed their heparin binding. Mutations targeted either the N-terminal or C-terminal half of the motif (first Half∗ and second Half∗, respectively), or all 8 basic residues within the HBM (HBM∗) (Fig. 1*D*). Recombinant proteins were expressed in human-derived Expi293 cells and purified by FLAG-tag affinity chromatography (Fig. S1, *A* and *B*). Heparin association was evaluated by heparin affinity chromatography with elution using a linear ionic gradient to 1.5 M NaCl (Figs. 1*E* and S1*C*). As expected, WT ERFE exhibited the strongest interaction, eluting at 800 mM NaCl. In contrast, both first Half∗ and second Half∗ eluted at substantially lower salt concentrations (520 mM and 541 mM NaCl, respectively), indicating a marked decrease in heparin affinity. Removing all basic residues in the HBM (HBM∗) strongly impaired heparin binding, and the mutant eluted at 418 mM NaCl.

In addition to directly assessing the contribution of basic residues within the HBM, we examined whether disruption of structural elements adjacent to the HBM affected heparin affinity. Notably, disruption of the collagen-like repeat (CLR∗) or combined disruption of the CLR and cysteines (CLR∗Cys∗) resulted in reduced heparin affinity (Figs. 1*E* and S1*D*). In contrast, mutation of the cysteines alone (Cys∗) or deletion of the ligand binding domain (ΔLBD) produced elution profiles similar to WT ERFE, indicating that these features do not meaningfully contribute to heparin affinity (Fig. S1*D*). Although both CLR∗ and CLR∗Cys∗ displayed similar reductions in heparin affinity, CLR∗Cys∗ was used for subsequent experiments because it was substantially easier to produce.

Next, we directly assessed heparin affinity using bio-layer interferometry (BLI). Biotinylated mixed-molecular weight (MMW) heparin was immobilized on streptavidin (SA)-coated sensor tips, and binding of WT ERFE and mutant proteins was measured over a concentration range of 3.75 to 240 nM. A representative curve at 240 nM ERFE is shown (Fig. 1*F*), with the complete 2-fold serial dilution series presented elsewhere (Fig. S2, *A*–*E*). Although the relative hierarchy from the heparin chromatography results was preserved, differences between WT ERFE and the mutants were far more pronounced. Little to no heparin binding was observed for the first Half∗, second Half∗, or HBM∗ mutants. Additionally, the CLR∗Cys∗ ERFE mutant was severely deficient in heparin affinity, despite containing a native HBM.

Next, we examined whether alterations in heparin affinity affected ERFE-mediated inhibition of BMP ligands. An osteoblast-derived, BMP-responsive luciferase reporter cell line.

(BRITER) was used to quantify signaling as ERFE was titrated against a constant concentration of BMP6 (Fig. 1*G*) (37). Notably, mutants with reduced heparin affinity exhibited corresponding decreases in BMP6 inhibition, with HBM∗ ERFE displaying >35-fold less inhibition than WT ERFE. Interestingly, previous work showed that CLR∗Cys∗ ERFE, which has lower heparin affinity, maintains full inhibition of BMP6 (14). Together, these findings suggest that the heparin-binding motif itself, rather than the heparin affinity of unbound ERFE, is important for BMP antagonism.

### The ERFE:BMP6 complex maintains a higher affinity for heparan sulfate than either component alone

**Figure 2:**
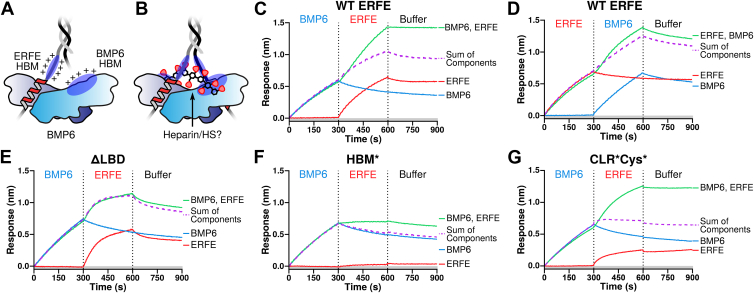
**Heparan sulfate facilitates the formation of an ERFE:BMP6:heparin complex with higher heparin affinity than either component alone.***A*, schematic showing heparin binding motifs of ERFE and BMP6 in the hypothesized complex. Only one HBM is highlighted in each protein for clarity. *B*, proposed heparin/HS binding site that could simultaneously interact with BMP6 and ERFE. *C*, SA-biotin heparin BLI tips were bound to BMP6 (*blue*) or ERFE (*red*) and summed together to create a theoretical curve (*purple*) that would represent independent binding. This was compared to sequential binding of BMP6 and then ERFE (*green*). *D*, the same experiment was performed, but the order was reversed, with ERFE first, followed by BMP6. *E-G*, BMP6 was bound to the BLI tips first, then the specified ERFE mutant. All BLI data shown are representative of n = 3 independent experiments.

We previously reported that the ERFE ligand binding domain (LBD) associates with the type I receptor binding site of BMP6. Based on this model, ERFE LBD binding positions its HBM adjacent to the known HBM of BMP6 (Fig. 2*A*) (27); however, having multiple HBMs in close proximity would be energetically unfavorable due to electrostatic repulsion. We hypothesized that heparin/HS could occupy this positively charged pocket and bridge both HBMs, thereby stabilizing the ERFE:BMP6 complex (Fig. 2*B*). Direct measurement of heparin affinity of pre-formed ERFE:BMP6 was not feasible, as the complex is insoluble under the neutral conditions required for heparin study. Interestingly, we observed that addition of heparin with chain lengths of dp10 (degree of polymerization 10, or a 10-saccharide chain) or greater was sufficient to maintain the solubility of the BMP6:ERFE complex (Fig. S3*A*). In contrast, BMP6 alone is mostly insoluble at neutral pH and its solubility is not improved upon the addition of heparin alone (Fig. S3*B*)

To circumvent these solubility limitations, we used BLI to analyze sequential binding of ERFE and BMP6 to heparin. First, we measured individual binding of ERFE or BMP6 to SA-biotin-heparin sensor tips (Fig. 2*C*, Red, Blue). The two response curves were summed to create a theoretical association and dissociation curve for additive, but independent binding of ERFE and BMP6 (Purple, dashed). We compared this theoretical curve to the sequential addition of BMP6 followed by ERFE. This comparison showed sequentially bound proteins had markedly enhanced association and extremely slow dissociation when compared to theoretical independent binding events (Green). This suggests that ERFE has substantially higher affinity for BMP6:heparin *versus* heparin alone.

To deconvolute these interactions and determine if synergistic binding was specifically driven by ERFE binding to the BMP6:heparin complex, we performed several follow-up experiments. When the experiment was performed in reverse, binding ERFE to heparin followed by the addition of BMP6, the same synergistic association was not observed (Fig. 2*D*). Next, we tested whether ΔLBD ERFE, which lacks the BMP-binding segment, could bind the BMP6:heparin complex. The observed response matched the theoretical sum of the individual binding curves, confirming that the enhanced association depends on the direct interactions of ERFE with BMP6 (Fig. 2*E*). Moreover, sequential binding of BMP6 followed by HBM∗ ERFE still showed synergistic binding, albeit to a much lesser extent than WT ERFE (Fig. 2*F*). This suggests ERFE:BMP6 interactions alone, in the absence of heparin binding, are insufficient to drive high-affinity complexation. In contrast, the CLR∗Cys∗ mutant, which exhibits reduced heparin affinity, displayed strong synergistic binding to heparin:BMP6 similar to WT ERFE (Fig. 2*G*). While deficient in heparin association, the presence of the LBD and HBM in this mutant enabled high levels of complex formation. Consistent with WT ERFE, synergistic binding was not observed for any mutant if ERFE was bound to heparin before BMP6 (Fig. S4, *A*–*C*). Together, these data demonstrate a defined preferred order of binding wherein ERFE engages the BMP6:heparin complex with greater affinity than either component alone.

### Computational modeling and molecular dynamics support a model where heparan sulfate bridges the interface of BMP6 and ERFE

**Figure 3:**
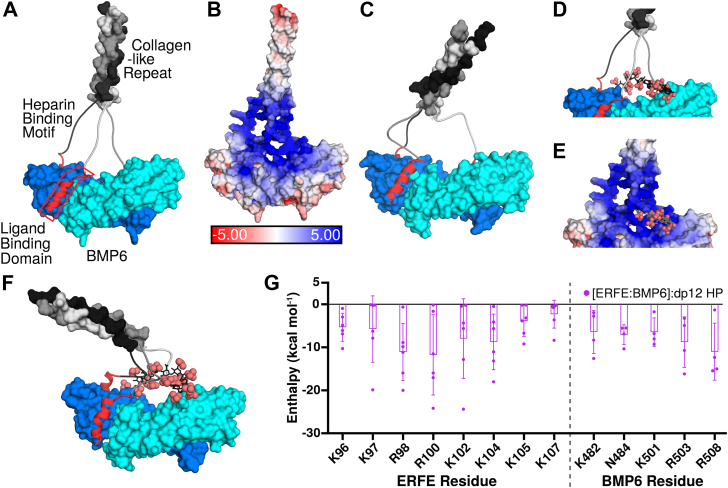
**Molecular dynamics (MD) simulations reveal that docked heparan sulfate (HS) can simultaneously interact with ERFE and BMP6.***A*, ERFE trimer:BMP6 model generated using AlphaFold 3, with conserved elements indicated *via* text. *B*, electrostatic surface potential of the complex generated using APBS electrostatics, with the scale in units of *k*_*B*_*T/e*_*c*_. *C*, representative 300 ns MD simulation of the alphafold-generated model. Heparan sulfate (HS) was docked into the positive pocket, shown in (*D*) cartoon and (*E*) charge map. In all models, HS is represented using sticks with *red* spheres to highlight the sulfate groups. Note that APBS-generated electrostatics do not account for docked small molecules. *F*, representative 300 ns MD simulation of the same starting model with the addition of HS. *G*, MMGBSA per-residue decomposition of the interaction enthalpy between the indicated residues in the composite HBM and HS. Each data point represents the enthalpy of the indicated residue in a chain in each of two independent experiments.

To further examine the interactions of HS with BMP6 and ERFE, we performed a series of molecular dynamics (MD) simulations. First, we generated a hypothetical model using AlphaFold 3, consisting of a BMP6 dimer and a truncated ERFE trimer containing the ligand binding domain (LBD), HBM, and collagen-like repeat (CLR) (Figs. 3*A* and S5*A*). As expected, this model contained an extremely positive patch at the BMP6:ERFE interface (Fig. 3*B*). A 300 ns simulation of the complex showed that the HBMs of ERFE and BMP6 exhibited strong electrostatic repulsion and rapidly reached a high RMSD state where all HBMs were positioned further away from each other than the starting model (Figs. 3*C* and S5*B*). In contrast, both the ERFE LBD:BMP6 interactions and CLR trimerization remained unchanged throughout the simulation, suggesting these interactions are stable. We observed that two ERFE LBDs formed strong interactions with the BMP6 type I receptor sites (Δ*H* = −94.4 kcal/mol each). Because the BMP dimer only contains two such sites, the third ERFE LBD formed interactions with nonpolar residues in the type II receptor site with substantially lower enthalpy (Δ*H* = −24.4 kcal/mol). Taken together, this supports our previously established hypothesis that ERFE primarily interacts with BMP6 through a pair of LBD:type 1 receptor site pairings and suggests the positively charged HBMs may repulse each other in the absence of a stabilizing force.

Next, we docked dp12 highly sulfated (IdoA(2S)-GlcNS(6S)) HS into the ERFE:BMP6 starting model using HDOCK (Fig. 3, *D* and *E*). The chain length was selected because it is sufficient to span the composite HBM and represents the biologically relevant length shown to bind BMP2 (24). The docking position was chosen based on proximity to the established BMP6 HBM, while excluding non-physiological models dominated by interactions with the chain termini. In a 300 ns simulation, we observed that HS formed extensive hydrogen bonds with the HBMs of ERFE and BMP6 to colocalize them, in contrast to our previous simulations. (Figs. 3*F* and S5*C*). We observed a total of 54 and 57 hydrogen bonds in our two replicates, split between BMP6 (24, 19) and ERFE (30, 38). While their total numbers were similar, they were distributed among different residues interacting with different sulfations in each MD simulation. In both models, per-residue MMGBSA (molecular mechanics-generalized born surface area) energy decomposition revealed that residues in both the ERFE and BMP6 HBM contributed favorably to HS interaction enthalpy (Fig. 3*E*). Notably, the residues did not contribute favorably to ERFE:BMP6 interactions in simulations lacking HS (Fig. S5*D*). Collectively, these simulations support that a single HS molecule can simultaneously engage the HBM of both ERFE and BMP6, providing an electrostatic bridge that stabilizes the inhibitory complex.

### The ERFE:Heparin:BMP6 Complex is a Discrete, High Molecular Weight Assembly

**Figure 4:**
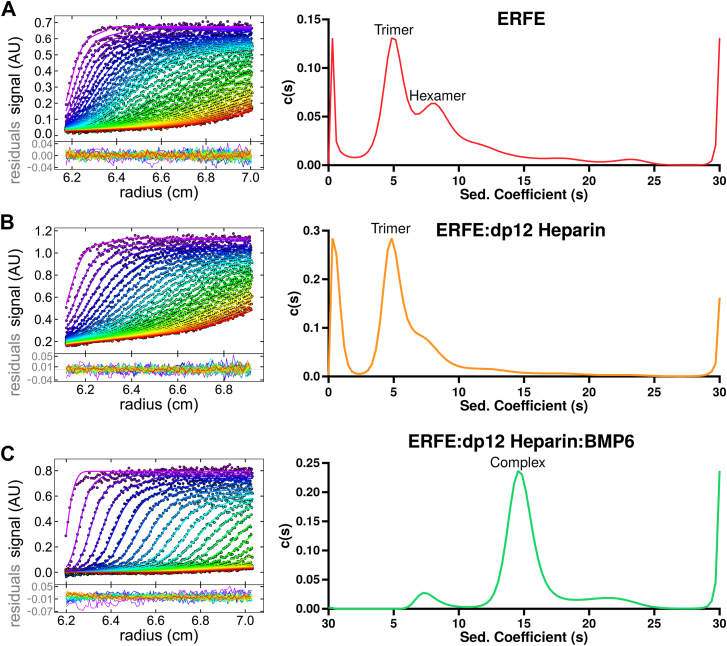
**The ERFE:BMP6:heparin complex is a distinct, high order species.** Sedimentation velocity analytical ultracentrifugation was used to determine the sedimentation coefficient of (*A*) ERFE, (*B*) ERFE:dp12 heparin, and (*C*) ERFE:dp12 heparin:BMP6. Data-fit-residual plots generated by GUSSI are shown to the *left*, while the c(s) fit of each experiment is shown to the *right* with species labeled based on estimated molecular mass.

Next, we wanted to characterize the biophysical properties of the ERFE:BMP6 complex in solution. To investigate this, we used sedimentation velocity analytical ultracentrifugation to examine ERFE, ERFE:dp12 heparin, and ERFE:dp12 heparin:BMP6 complexes in solution (ERFE:BMP6 was excluded due to its insolubility). We used dp12 heparin to form the ERFE:BMP6 complex, as it is sufficient to maintain complex solubility in solution. Additionally, its heavy sulfation resembles the highly sulfated HS identified in hepatocytes while also being more readily available than HS for experimental use (38, 39). WT ERFE sedimented as previously reported, exhibiting a high frictional ratio (*f/f*_*0*_ *=* 2.06), typical of an elongated, partially unstructured protein. We observed a major peak corresponding to a trimer (s_*20,w*_ = 5.23 ± 0.08, *M*_app_ = 164 kDa) and a minor peak corresponding to a hexamer (s_*20,w*_ = 9.15 ± 0.20, *M*_app_ = 403 kDa) (Fig. 4*A*). ERFE:dp12 heparin exhibited a similar frictional ratio (*f/f*_*0*_ *=* 2.14) and sedimentation coefficient (s_*20,w*_ = 4.82 ± 0.19, *M*_app_ = 154 kDa), but the abundance of higher-order oligomers was reduced (Fig. 4*B*).

In sharp contrast, ERFE:dp12 heparin:BMP6 sedimented with a much smaller frictional ratio (*f/f*_*0*_ *=* 1.08) with a much higher sedimentation coefficient (s_*20,w*_ = 14.59 ± 0.13, *M*_app_ = 299 kDa) (Fig. 4*C*). Generally, a large decrease in frictional ratio suggests a conformational change towards a more globular form. However, the frictional ratio of a solvated sphere is 1.2, making this interpretation unlikely. Rather, microheterogeneity may be present in the sample due to different orientations and quantities of heparin chains bound to each complex. This could lead to improper fitting of data and an underestimation of the frictional ratio and true molecular mass. As such, the dramatic shift in the sedimentation coefficient is consistent with the formation of a discrete complex with multiple HS binding sites containing multiple ERFE trimers and BMP6 dimers. The formation of this higher-order complex provides a structural basis for the avidity and cooperative binding observed in our biochemical assays.

### The ERFE:BMP6 complex is localized to the cell surface in a heparan sulfate-dependent and ligand-dependent manner

**Figure 5:**
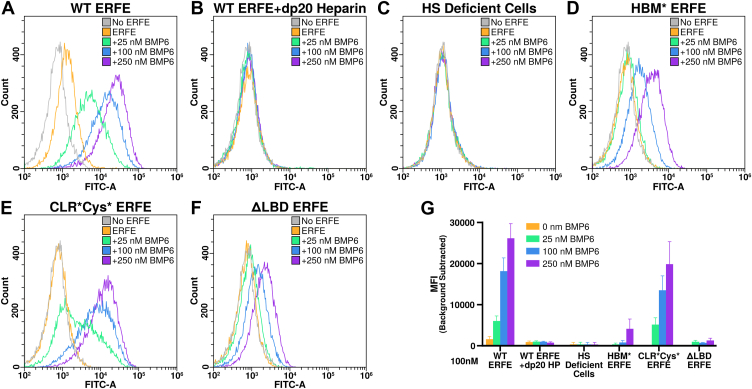
**The ERFE:BMP6 complex is localized to the cell surface in a HS- and ligand-dependent manner.***A*, CHO-K1 cells were gently trypsinized and incubated with 100 nM ERFE and the listed concentration of BMP6, before probing for ERFE *via* anti-flag immunodetection and analysis *via* fluorescence detection flow cytometry. This was repeated with (*B*) the addition of 20:1 dp20 heparin:ERFE. *C*, HS N-sulfotransferase-deficient CHO-K1 cells which lacked all HS sulfation, (*D*) HBM∗ ERFE, (*E*) secondary structure-disrupting CLR∗Cys∗ ERFE, and (*F*) ΔLBD ERFE. All histograms are representative of n = 3 biological replicates. *G*, quantification of mean fluorescence intensity (MFI) of n = 3 replicates on a linear scale where error bars represent the S.E.M.

Heparin, used in our *in vitro* biophysical experiments, is heavily sulfated along its entire length, providing numerous ligand-binding sites. The extracellular matrix, in contrast, contains heparan sulfate (HS), which contains short, sulfated regions interspersed with long unsulfated stretches. This structural heterogeneity makes quantitative biophysical analysis challenging. To test whether the ERFE:BMP6 complex strongly binds to HS, we used the cell surface of immortalized cells as a surrogate. Here, we selected CHO-K1 cells and a matched cell line deficient in HS N-sulfotransferase, which lack all HS sulfation patterns (pgsE-606) (40). To quantify cell surface association, CHO-K1 cells were incubated with ERFE and varying BMP6 concentrations at 4 °C, and bound ERFE was quantified by fluorescence-based flow cytometry. ERFE alone (100 nM) yielded only weak fluorescence, whereas addition of 250 nM BMP6 caused an approximately 20-fold increase in cell surface fluorescence (Fig. 5*A*).

To confirm that cell surface association was mediated by heparan sulfate, we repeated the experiment with excess dp20 heparin. Co-incubation of either ERFE or ERFE:BMP6 complex with exogenous heparin abolished all cell surface binding (Fig. 5*B*). Similarly, neither ERFE nor the ERFE:BMP6 complex associated with the matched cell line, which lacked HSPG sulfation (Fig. 5*C*) (40). Analysis of HBM∗ ERFE revealed little binding to WT cells, although a small ligand-dependent increase was detectable (Fig. 5*D*). Interestingly, CLR∗Cys∗ ERFE did not bind to WT cells in isolation but bound as strongly as WT ERFE in the presence of BMP6 (Fig. 5*E*).

All experiments were replicated at 500 nM ERFE, demonstrating that higher concentrations of ERFE could not rescue deficiencies in cell surface binding (Fig. S6, *A–F*). Finally, ΔLBD ERFE failed to exhibit ligand-dependent cell surface association (Fig. 5*F*), confirming that direct BMP6 interactions are required. Together, these data indicate that ERFE cell surface binding depends on coordinated complex formation with cell-surface HSPGs and BMP6 (Fig. 5*G*), requiring both the ERFE ligand binding domain (LBD) and HBM and is not driven by the intrinsic HS affinity of ERFE alone.

### Various ligands with different charge Distributions are unable to promote ERFE association with the cell surface

**Figure 6:**
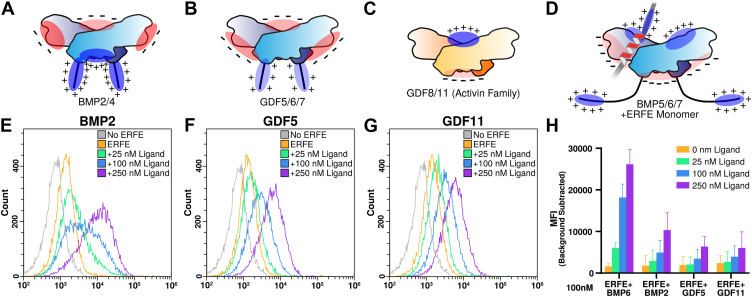
**Ligands without the BMP6 HBM are unable to drive strong cell surface association.***A-C*, established charged regions of the indicated ligands are shown for comparison with (*D*) BMP6 in simplified schematics. CHO-K1 cells were again used to quantify ERFE association with the cell surface by titrating in (*E*) BMP2, (*F*) GDF5, or (*G*) GDF11. All histograms are representative of n = 3 biological replicates. *H*, quantification of MFI of n = 3 replicates on a linear scale where error bars represent the S.E.M.

Previous studies have demonstrated that ERFE binds with high affinity to BMP2 and BMP6 in biochemical assays, such as SPR (3). However, in cell-based assays, ERFE inhibits BMP6 >30-fold more potently than BMP2 (14). Given our findings that heparin/HS interactions are critical for BMP6 antagonism, we postulated that they might also contribute to ligand selectivity. One possible explanation for ERFE specificity lies in the distinct location of heparin-binding regions across ligands. BMP6 binds heparin/HS on the top surface of the ligand, facing away from the cell surface when in complex with its receptors, while BMP2/4 and GDF5/6/7 feature negatively charged regions in this location (Fig. 6, *A*, *B* and *D*) (24, 27, 41, 42). Instead, BMP2 contains an extensive HBM on the bottom of the ligand, facing the cell surface. Many other TGF-β family ligands—including TGF-β, activin A, GDF8, and GDF11—do not appreciably bind heparin (16, 32, 43). Although GDF11 contains a top-facing positively charged patch reminiscent of BMP6 (Fig. 6*C*), it is not inhibited by ERFE; however, this region can synergize with the HBM of the extracellular antagonist follistatin to enhance heparin affinity (14, 32).

To determine whether ERFE exhibits ligand-dependent cell surface association with other TGF-β ligands, we performed cell surface binding experiments with BMP2, GDF5, and GDF11. Despite its stronger HBM and higher ECM affinity, BMP2 induced only weak ligand-dependent ERFE localization to the cell surface (Fig. 6*E*) (25). GDF5, which is known to have minimal interactions with the cell surface, and the structurally unrelated GDF11 both showed even weaker ligand-dependent association (Fig. 6, *F* and *G*). Compared to BMP6, all three ligands were markedly deficient in their ability to drive ERFE localization to the cell surface (Fig. 6*H*). Together, these findings indicate that ERFE does not bind heparin-binding ligands indiscriminately, but instead preferentially targets the BMP6:HS complex. Ligand-specific HBM positioning and ECM topology therefore appear to be critical determinants of ERFE selectivity and functional antagonism.

## Discussion

Erythroferrone (ERFE) is the erythroid-derived regulator of iron homeostasis (1). In response to a blood loss event, developing erythroblasts produce ERFE to upregulate serum iron levels and enable their rapid maturation (1). To facilitate this, ERFE travels from the place of stress erythropoiesis, in the bone marrow or spleen, to the liver, where it inhibits BMP-dependent hepcidin induction (7, 44). Notably, ERFE does not impact BMP signaling elsewhere in the body, even when moderately overexpressed, suggesting the existence of a molecular mechanism that constrains its antagonism (4).

Previous studies identified that ERFE contains a positively charged heparin-binding motif (HBM) and its deletion reduced BMP inhibition (33). However, the mechanistic roles of the ERFE HBM and HS in BMP inhibition were unclear. In this publication, we demonstrate that HS simultaneously engages the HBMs of both BMP6 and ERFE to facilitate the formation of high-order complexes. This suggests that interactions with HS cluster the ERFE:BMP6 complex on the cell surface and contribute to ligand selectivity of ERFE. Taken together, our findings support a mechanism that is fundamentally distinct from previously characterized extracellular antagonists of the TGF-β family.

First, ERFE is a circulating antagonist that must exclusively antagonize BMP signaling in the liver; inhibition of BMP7 in the kidney, for example, could disrupt its well-established renoprotective effects (45). Our cell-surface binding experiments suggest ERFE weakly associates with cell surface HSPGs in an epithelial cell line. This stands in contrast to our chromatography and BLI experiments, which showed tight association with heparin, a more heavily sulfated polysaccharide. Importantly, HS sulfation varies considerably throughout the body and is heaviest in the liver, where sulfation of certain regions closely resembles heparin rather than typical HS (38, 39). The presence of basement-membrane-specific proteins, like perlecan, has been identified *in vivo* in the perisinusoidal space, alongside glypicans and syndecans (46). In contrast, HSPGs in the kidney are undersulfated when compared to the rest of the body (38). This sulfation gradient suggests that ERFE circulates with minimal cell surface interactions in most tissues but may be preferentially retained in the liver owing to its highly sulfated HSPG environment.

Second, ERFE must target class II BMPs to prevent further off-target effects. Our data strongly suggest that ERFE preferentially binds to the BMP6:heparin/HS complex. In isolation, the HBMs of ERFE and BMP6 may repulse and limit complex formation. Our MD simulations suggest that HS can counteract those repulsive forces to stabilize the complex in solution by simultaneously interacting with the HBMs of ERFE and BMP6. Class II BMPs are the only ligands with an HBM proximally located to the complex interface, which may explain why they are the only BMPs potently inhibited by ERFE and able to cause cell-surface localization of the complex. The BMP2/6 heterodimer, presently hypothesized to be the primary regulator of hepcidin *in vivo*, would also be able to form BMP:HS complexes in a similar manner to class II BMPs (19, 47). This may explain why ERFE inhibits BMP2/6 as potently as BMP6, despite not potently inhibiting BMP2 (19).

Finally, ERFE is the only trimeric antagonist of any TGF-β family ligand. Our analytical ultracentrifugation data strongly suggests that, in the presence of heparin/HS, ERFE forms a terminal high-order complex with BMP6. This was expected, as there is a stoichiometric mismatch between trimeric ERFE and dimeric BMP6. As observed in our MD simulations, ERFE is only able to form two high-affinity interactions with a BMP6 dimer; facilitated by the binding of the ERFE ligand-binding domain (LBD) to a type I receptor binding site of BMP6. This leaves an third, unbound LBD able to bind to another BMP6 ligand and form a high-order complex. This could give rise to 2 potential terminal complexes. We would expect it to contain either two ERFE trimers and three BMP6 ligands (six LBD and type I receptor site pairings) or four ERFE trimers and six BMP6 ligands (twelve pairings). While we were unable to conclude the precise stoichiometry of the complex, it contains multiple HS binding sites, which would give it avidity for the cell surface. Thus, the formation of high-order complexes could explain why ERFE alone exhibits minimal binding to the cell surface, but the ERFE:BMP6 complex is strongly localized to it in an HSPG-dependent manner. This complex could then be primed for endocytosis and degradation, consistent with other proteins that tightly associate with HSPGs (23, 32).

ERFE is not the only antagonist to interact with HSPGs and the ECM; the BMP antagonists noggin and gremlin1/2 strongly associate with the ECM and the cell surface in a ligand-independent manner (30, 31). This creates ligand-antagonist signaling gradients essential for embryonic patterning (23, 30, 48). Thus, while the HBMs of these antagonists are essential for their biological role, they are believed to be dispensable for BMP ligand binding (31). Other antagonists, like follistatin288, are able to form composite HBMs spanning both the ligand and the antagonist (32). These interactions facilitate the clearance of the terminally sequestered antagonist:ligand complex from the serum, but, again, are not directly involved in ligand binding and inhibition (32, 49). Thus, ERFE represents a new mode of TGF-β family antagonism: its HBM is required for activity and proper complex formation and may be responsible for both tissue targeting and ligand specificity.

**Figure 7:**
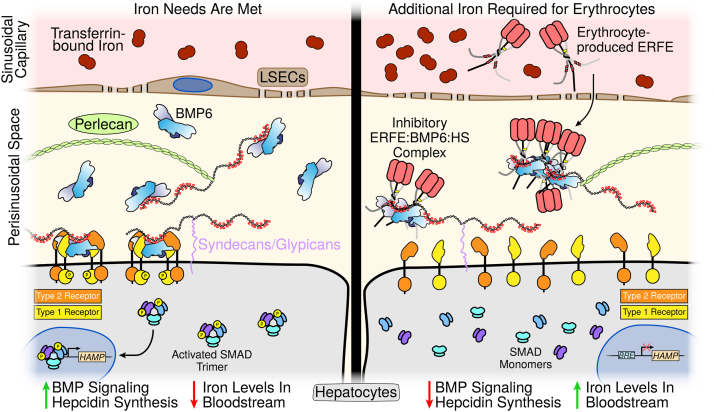
**Proposed biological mechanism for ERFE inhibition of BMP ligands.** Under basal conditions, sufficient iron levels are sensed by liver sinusoidal endothelial cells (LSECs), which leads to BMP6 upregulation. BMP6 binds to heparan sulfate proteoglycans, like syndecans or glypicans, on the cell surface of hepatocytes, initiating a signal by binding to 2 type I and 2 type II receptors. This leads to SMAD phosphorylation and upregulation of hepcidin (*HAMP*) mRNA *via* the BMP Responsive Element (*BRE*) promoter. Translated hepcidin is then secreted and negatively regulates gut and tissue iron export to the bloodstream. When erythropoiesis is ongoing, erythroblasts produce ERFE, which coordinates with heparan sulfate proteoglycans on the hepatocyte cell surface and in the perisinusoidal space to form high-order complexes with BMP6. These inhibitory complexes are stable and have a high affinity for heparan sulfate, sequestering the ligand. This prevents hepcidin induction and allows for high serum iron levels to facilitate rapid generation of hemoglobin required for erythrocyte maturation.

Therefore, we propose a biological model for ERFE regulation of BMP signaling in the liver (Fig. 7). Under basal conditions, liver sinusoidal endothelial cells sense transferrin-bound and unbound iron in the bloodstream and produce BMP6 in response (5, 6, 8). BMP6 coordinates with cell surface proteoglycans on hepatocytes to upregulate hepcidin, which inhibits iron import from the gut and export from tissues into the bloodstream (8, 26, 27, 50, 51). When the body has a higher requirement for iron after a blood loss event, developing red blood cells produce ERFE (1). ERFE has low affinity for the ECM and travels from the site of stress erythropoiesis to the perisinusoidal space, which is enriched for highly sulfated HSPGs, like glypicans, syndecans, and perlecan (46, 52). Here, ERFE binds to HSPG-associated BMP6 ligands to form high-order complexes and terminally sequester them. This prevents hepcidin induction, increasing serum iron levels and allowing the body to rapidly regenerate red blood cells (1, 44).

Collectively, this study sheds light on the integration of heparin/HS in the molecular mechanism of BMP inhibition by ERFE. The results show that ERFE is a “matrix-assisted” antagonist, which helps explain its narrow physiological footprint. Presently, the development of therapeutic biologics is exploring strategies to target different tissues and cell types. Therefore, a better understanding of this novel targeting strategy might provide new opportunities to develop biologics that selectively target BMP molecules in highly sulfated environments like the liver, while sparing essential BMP function in other peripheral tissues.

## Experimental procedures

### Recombinant ligands and detection antibodies

BMP2 and GDF5 were produced as previously described (28). Mature GDF11 was a generous gift from Elevian. BMP6 was a generous gift from Dr Slobodan Vukicevic (Genera Research). In western blots, ERFE was detected using DYKDDDDK Epitope Tag HRP-conjugated antibody (1:5000, R&D, Cat:HAM85291) and BMP6 was detected using anti-BMP6 (1:1000, R&D, Cat:AF6325) followed by HRP-conjugated anti-sheep (1:1000, R&D, Cat:HAF016).

### Protein production and purification

ERFE production and purification were performed as previously described (14). In short, an N-terminally flag-tagged human ERFE construct (residues 44–354) in the mammalian expression vector pcDNA3.1, generously provided by Dr Tomas Ganz (UCLA), was used as a starting point for all constructs. Purified plasmid was transfected with polyethyleneimine into Expi293 cells, and conditioned media was collected on day 3. Constructs with high affinity for heparin were applied to a HiPrep Heparin FF affinity column and eluted with a NaCl gradient in HEPES (pH 7.5). Constructs with weaker heparin affinity (elution <650 mM NaCl) were applied to Pierce anti-DYKDDDDK resin (Cat:A36801) and eluted with 100 mM glycine (pH 2.5) and neutralized with 500 mM HEPES (pH 7.5), 150 mM NaCl. The resulting peaks were dialyzed into 20 mM HEPES (pH 7.5), 150 mM NaCl, 5 mM CaCl_2_, concentrated to 1 mg/ml using Pierce PES protein concentrators (Cat:88517) and either used immediately or flash frozen and stored at −80 °C.

### Analytical heparin affinity chromatography

100 μg of purified WT or mutant ERFE was loaded onto a 1 ml HiTrap Heparin HP column (Cytiva, Prod. 17040601) using an AKTA Pure chromatography system (Cytiva). Each run was eluted with an identical linear NaCl gradient (0–1.5 M in 20 mM HEPES (pH 7.5), 5 mM CaCl_2_) over 20 ml at 4 °C, with the representative conductivity trace from 0 to 1 M NaCl shown.

### Heparin biolayer interferometry (BLI)

Mixed molecular weight heparin (Sigma-Aldrich, Cat:H3393) was conjugated to biotin as previously described by coupling the reducing terminus with EZ-link NHS-PEG_4_-biotin (Thermo Scientific, Cat:21330) following reductive amination with NaCNBH_3_ (Thermo Scientific, Cat:44892) (53). After repeated dialysis into sterile water, the biotinylated heparin was lyophilized. BLI was performed using an Octet RED96 (ForteBio) and Streptavidin biosensors (Sartorius, Cat:18-5019). Tips were coupled with 1 mg/ml biotinylated heparin and blocked with 2 mg/ml PEG_4_-biotin. As differences in coupling density were observed, all curves placed on the same graph were from the same tip. To ensure that results were not due to differences in coupling density, each independent replicate was performed using a separately coupled tip. All experiments were performed using 20 mM HEPES pH 7.5, 250 mM NaCl, 4 mM EDTA, and 0.005% Tween-20 at 22 °C, which limited nonspecific binding without disrupting ERFE association to heparin. Tips were regenerated using 2 M NaCl.

### Luciferase reporter assay

A BMP-responsive luciferase reporter osteoblast cell line (BRITER, RRID:CVCL_0P40), provided by Dr Amitabha Bandyopadhyay (Indian Institute of Technology), was used to measure BMP activity as previously reported (14, 37). Briefly, cells were grown overnight in α-minimal essential medium with 10% (v/v) FBS and 100 μg/ml hygromycin B in a 96-well plate at 37 °C in 5% CO_2_. The medium was replaced with Dulbecco's modified Eagle's medium (DMEM), and cells were starved for 5 h. Inhibition assays were performed by titrating ERFE in DMEM and adding a constant ligand concentration. The media was replaced with ligand or ligand plus inhibitor in DMEM and incubated at the same conditions for 3 h before cells were lysed and luminescence was recorded using a BioTek Synergy H1 Microplate Reader. Inhibitory curves were normalized using the ligand alone as 100% signal. Curve fits and IC_50_ values were generated using GraphPad Prism. All IC_50_ values were calculated using a variable hill slope. Each experiment consisted of three technical replicates for each concentration and was repeated separately three times. Error bars on graphs represent the SD of the three technical replicates from the representative sample. Reported IC_50_ are the average of three biological replicates ± SEM.

### Solubility analysis

ERFE and BMP6 were combined at a 4:1 M ratio on ice (0.5 μg ERFE, 0.125 μg BMP6) with a 1:3 ratio of ERFE:Heparin where indicated, and PBS was added to bring the total volume to 10 μl. Chemically defined heparin (dp4-dp20) was the generous gift from the lab of the late Dr Robert Lindhardt (Rensselaer Polytechnic Institute). After mixing, samples were incubated at room temperature for 5 min before centrifugation at 10k rpm at 4 °C for 10 min 7 μl of supernatant was removed and separated by SDS-PAGE using a 4 to 20% gradient (Bio-Rad, Cat:4568095) under reducing conditions before transferring to PVDF membrane and analysis by Western blot.

### Molecular dynamics (MD) simulations

BMP6 and ERFE structures, as well as their complex, were obtained by AlphaFold3 modelling (54), while the initial positions of heparan sulfate dp12 were generated using the HDOCK server (55). Unstructured protein regions modelled with low confidence were not included in the final AlphaFold3 model generated prior to docking and MD simulations; thus, only residues 410 − 513 of BMP6 and 76 − 125 of ERFE are included. Protonation states of ionizable amino acid residues were estimated using PROPKA 3.1 (56). Proteins were solvated in 20 Å octahedral boxes, neutralized with corresponding number of counterions, and parametrized with the AMBER ff14SB force field for proteins and the TIP3P model for water (57). Systems were subjected to geometry optimization in AMBER22 with periodic boundary conditions applied in all directions (58). The optimized systems were gradually heated from 100 to 298 K over 1 ns under constant volume and then equilibrated for 7 ns at a constant pressure, followed by production MD simulations of 300 ns without constraints. Each simulation was performed in duplicate. Trajectory processing and data analyses were carried out with the CPPTRAJ module implemented in AMBER (59). Interaction enthalpies were calculated from 5000 frames extracted from the final 50 ns of each replicate using the MM-GBSA protocol and decomposed into *per-residue* contributions (60).

### Sedimentation velocity analytical ultracentrifugation (SV-AUC)

Protein samples were used immediately after dialysis and were never frozen. Sedimentation velocity experiments were performed using a Beckman Optima XL-I analytical ultracentrifuge (Beckman Coulter), An60-Ti rotor, and absorbance optics. Samples at 0.25 mg/ml were loaded into Beckman Coulter analytical ultracentrifugation sample cells with 12-mm optical path two-channel centerpieces, with matched buffer in the reference sector and centrifuged at 20 k rpm. Absorbance was measured at 230 nm to maximize signal, and the experiments were performed at 20 °C. SEDFIT was used to analyze the data using a continuous c(s) model at a resolution of 150, first fit using Marquardt Levenberg and then Simplex Algorithm (61). c(s) values at the minimum and maximum sedimentation coefficients were reduced to the value of the largest sample peak to more clearly show observed ERFE states. The reported sedimentation coefficient values (± standard deviation) were identified by fitting the data with a confidence level of 0.5, without regularization, and the major peaks corresponding to the regularized data were integrated. M_app_ was then calculated using the sedimentation coefficient and frictional ratio using the calculator function on SEDFIT. Data-Fit-Error plots were generated using GUSSI with default settings, showing every third curve and third data point (62).

### Flow cytometry

CHO-K1 cells (RRID:CVCL_0214) and heparan sulfate-deficient CHO-K1 cells (RRID:CVCL_4593) were grown in HAM/F12 media with 10% (v/v) FBS and further supplemented with 1 mM L-glutamine. Cells were prepared for flow cytometry by a brief incubation in 1× PBS with 1 mM EDTA before trypsinization with 0.025% trypsin + EDTA to ensure cell surface HSPGs were not degraded. Cells were counted and resuspended at 5 × 10^6^ cells/ml in FACS buffer (ice-cold PBS, 1% BSA, 0.1% sodium azide). 5 × 10^5^ cells were added to a 96-well v-bottom plate for each sample. After pelleting at 200 × *g*, cells were resuspended in 20 μl FACS buffer containing the specified concentration of ERFE and ligand and were incubated on ice for 10 min. Cellular suspensions were diluted with 100 μl FACS buffer and washed twice with FACS buffer by sequential pelleting and resuspension. Cells were then incubated in 50 μl 1:1000 M.2 anti-flag antibody (1 mg/ml stock, a gracious gift of Dr Tom Seegar (University of Cincinnati)) diluted in FACS buffer for 30 min. After dilution with 100 μl FACS buffer, the cells were again washed. Cells were then resuspended and treated with 50 μl 1:1000 Alexa Fluor 488 anti-Mouse IgG (Invitrogen, Cat: A11001) for 30 min before washing using FACS buffer. After resuspension, 200 μl FACS buffer was added to the cells, which were then analyzed using a CytoFLEX Flow Cytometer (Beckman Coulter). Data analysis was performed using CytExpert (Beckman Coulter), and cells were gated for singlets. All experiments were performed in triplicate using different cell passages.

## Data availability

All data is contained within the paper.

## Supporting information

This article contains supporting information.

## Conflict of interest

The authors declare that they have no conflicts of interest with the contents of this article.

## References

[1] Kautz L., Jung G., Valore E.V., Rivella S., Nemeth E., Ganz T. (2014). Identification of erythroferrone as an erythroid regulator of iron metabolism. Nat. Genet..

[2] Kautz L., Jung G., Du X., Gabayan V., Chapman J., Nasoff M. (2015). Erythroferrone contributes to hepcidin suppression and iron overload in a mouse model of β-thalassemia. Blood.

[3] Arezes J., Foy N., McHugh K., Quinkert D., Benard S., Sawant A. (2020). Antibodies against the erythroferrone N-terminal domain prevent hepcidin suppression and ameliorate murine thalassemia. Blood.

[4] Coffey R., Jung G., Olivera J.D., Karin G., Pereira R.C., Nemeth E. (2022). Erythroid overproduction of erythroferrone causes iron overload and developmental abnormalities in mice. Blood.

[5] Fisher A.L., Wang C., Xu Y., Joachim K., Xiao X., Phillips S. (2022). Functional role of endothelial transferrin receptor 1 in iron sensing and homeostasis. Am. J. Hematol..

[6] Charlebois E., Fillebeen C., Presley J., Cagnone G., Lisi V., Lavallée V.-P. (2023). Liver sinusoidal endothelial cells induce BMP6 expression in response to non–transferrin-bound iron. Blood.

[7] Truksa J., Peng H., Lee P., Beutler E. (2006). Bone morphogenetic proteins 2, 4, and 9 stimulate murine hepcidin 1 expression independently of hfe, transferrin receptor 2 (Tfr2), and IL-6. Proc. Natl. Acad. Sci. U. S. A..

[8] Andriopoulos B., Corradini E., Xia Y., Faasse S.A., Chen S., Grgurevic L. (2009). BMP-6 is a key endogenous regulator of hepcidin expression and iron metabolism. Nat. Genet..

[9] Xiao X., Xu Y., Moschetta G.A., Yu Y., Fisher A.L., Alfaro-Magallanes V.M. (2023). BMP5 contributes to hepcidin regulation and systemic iron homeostasis in mice. Blood.

[10] Pauk M., Grgurevic L., Brkljacic J., Kufner V., Bordukalo-Niksic T., Grabusic K. (2015). Exogenous BMP7 corrects plasma iron overload and bone loss in Bmp6-/- mice. Int. Orthop..

[11] Galy B., Conrad M., Muckenthaler M. (2024). Mechanisms controlling cellular and systemic iron homeostasis. Nat. Rev. Mol. Cell Biol..

[12] Schanbacher C., Hermanns H.M., Lorenz K., Wajant H., Lang I. (2023). Complement 1q/Tumor necrosis factor-related proteins (CTRPs): structure, receptors and signaling. Biomedicines.

[13] Stewart A.N., Little H.C., Clark D.J., Zhang H., Wong G.W. (2020). Protein modifications critical for Myonectin/Erythroferrone secretion and oligomer assembly. Biochemistry.

[14] Mast E.M., Leach E.A.E., Thompson T.B. (2024). Characterization of erythroferrone oligomerization and its impact on BMP antagonism. J. Biol. Chem..

[15] Mörsdorf D., Knabl P., Genikhovich G. (2024). Highly conserved and extremely evolvable: BMP signalling in secondary axis patterning of Cnidaria and Bilateria. Dev. Genes Evol..

[16] Hinck A.P., Mueller T.D., Springer T.A. (2016). Structural biology and evolution of the TGF-β family. Cold Spring Harb. Perspect. Biol..

[17] Akiyama T., Raftery L.A., Wharton K.A. (2024). Bone morphogenetic protein signaling: the pathway and its regulation. Genetics.

[18] Gipson G.R., Goebel E.J., Hart K.N., Kappes E.C., Kattamuri C., McCoy J.C. (2020). Structural perspective of BMP ligands and signaling. Bone.

[19] Wang C.-Y., Xu Y., Traeger L., Dogan D.Y., Xiao X., Steinbicker A.U. (2020). Erythroferrone lowers hepcidin by sequestering BMP2/6 heterodimer from binding to the BMP type I receptor ALK3. Blood.

[20] Hirsh J., Anand S.S., Halperin J.L., Fuster V. (2001). Mechanism of action and pharmacology of unfractionated heparin. Arterioscler. Thromb. Vasc. Biol..

[21] Baytas S.N., Linhardt R.J. (2020). Advances in the preparation and synthesis of heparin and related products. Drug Discov. Today.

[22] Theocharis A.D., Skandalis S.S., Gialeli C., Karamanos N.K. (2016). Extracellular matrix structure. Adv. Drug Deliv. Rev..

[23] Sarrazin S., Lamanna W.C., Esko J.D. (2011). Heparan sulfate proteoglycans. Cold Spring Harb. Perspect. Biol..

[24] Smith R.A.A., Murali S., Rai B., Lu X., Lim Z.X.H., Lee J.J.L. (2018). Minimum structural requirements for BMP-2-binding of heparin oligosaccharides. Biomaterials.

[25] Kim M.G., Kim C.L., Kim Y.S., Jang J.W., Lee G.M. (2021). Selective endocytosis of recombinant human BMPs through cell surface heparan sulfate proteoglycans in CHO cells: BMP-2 and BMP-7. Sci. Rep..

[26] Irie A., Habuchi H., Kimata K., Sanai Y. (2003). Heparan sulfate is required for bone morphogenetic protein-7 signaling. Biochem. Biophys. Res. Commun..

[27] Denardo A., Elli S., Federici S., Asperti M., Gryzik M., Ruzzenenti P. (2021). BMP6 binding to heparin and heparan sulfate is mediated by N-terminal and C-terminal clustered basic residues. Biochim. Biophys. Acta BBA - Gen. Subj..

[28] Gipson G.R., Nolan K., Kattamuri C., Kenny A.P., Agricola Z., Edwards N.A. (2023). Formation and characterization of BMP2/GDF5 and BMP4/GDF5 heterodimers. BMC Biol..

[29] Heide F., Legare S., To V., Gupta M., Gabir H., Imhof T. (2022). Heparins mediate the multimer assembly of secreted Noggin. Protein Sci..

[30] Paine-Saunders S., Viviano B.L., Economides A.N., Saunders S. (2002). Heparan sulfate Proteoglycans retain noggin at the cell surface: a potential mechanism for shaping bone morphogenetic protein gradients∗. J. Biol. Chem..

[31] Kattamuri C., Nolan K., Thompson T.B. (2017). Analysis and identification of the Grem2 heparin/heparan sulfate-binding motif. Biochem. J..

[32] Cash J.N., Rejon C.A., McPherron A.C., Bernard D.J., Thompson T.B. (2009). The structure of myostatin:follistatin 288: insights into receptor utilization and heparin binding. EMBO J..

[33] Srole D.N., Jung G., Waring A.J., Nemeth E., Ganz T. (2023). Characterization of erythroferrone structural domains relevant to its iron-regulatory function. J. Biol. Chem..

[34] Crooks G.E., Hon G., Chandonia J.-M., Brenner S.E. (2004). WebLogo: a sequence logo generator. Genome Res..

[35] Cardin A.D., Weintraub H.J. (1989). Molecular modeling of protein-glycosaminoglycan interactions. Arterioscler. Off. J. Am. Heart Assoc. Inc.

[36] Muñoz E.M., Linhardt R.J. (2004). Heparin-Binding domains in vascular biology. Arterioscler. Thromb. Vasc. Biol..

[37] Yadav P.S., Prashar P., Bandyopadhyay A. (2012). BRITER: a BMP responsive osteoblast reporter cell line. PLOS ONE.

[38] Vongchan P., Warda M., Toyoda H., Toida T., Marks R.M., Linhardt R.J. (2005). Structural characterization of human liver heparan sulfate. Biochim. Biophys. Acta BBA - Gen. Subj..

[39] Lyon M., Deakin J.A., Gallagher J.T. (1994). Liver heparan sulfate structure. A novel molecular design. J. Biol. Chem..

[40] Bame K.J., Zhang L., David G., Esko J.D. (1994). Sulphated and undersulphated heparan sulphate proteoglycans in a Chinese hamster ovary cell mutant defective in N-sulphotransferase. Biochem. J..

[41] Seeherman H.J., Berasi S.P., Brown C.T., Martinez R.X., Juo Z.S., Jelinsky S. (2019). A BMP/activin A chimera is superior to native BMPs and induces bone repair in nonhuman primates when delivered in a composite matrix. Sci. Transl. Med..

[42] Nolan K., Kattamuri C., Rankin S.A., Read R.J., Zorn A.M., Thompson T.B. (2016). Structure of Gremlin-2 in complex with GDF5 gives Insight into DAN-Family-Mediated BMP antagonism. Cell Rep..

[43] Goebel E.J., Hart K.N., McCoy J.C., Thompson T.B. (2019). Structural biology of the TGFβ family. Exp. Biol. Med..

[44] Arezes J., Foy N., McHugh K., Sawant A., Quinkert D., Terraube V. (2018). Erythroferrone inhibits the induction of hepcidin by BMP6. Blood.

[45] Tsujimura T., Idei M., Yoshikawa M., Takase O., Hishikawa K. (2016). Roles and regulation of bone morphogenetic protein-7 in kidney development and diseases. World J. Stem Cells.

[46] Roskams T., Moshage H., De Vos R., Guido D., Yap P., Desmet V. (1995). Heparan sulfate proteoglycan expression in normal human liver. Hepatol. Baltim. Md..

[47] Xiao X., Dev S., Canali S., Bayer A., Xu Y., Agarwal A. (2020). Endothelial Bmp2 knockout exacerbates hemochromatosis in hfe knockout mice but not Bmp6 knockout mice. Hepatol. Baltim. Md..

[48] Krause C., Guzman A., Knaus P.N. (2011). Int. J. Biochem. Cell Biol..

[49] Walker R.G., Kattamuri C., Goebel E.J., Zhang F., Hammel M., Tainer J.A. (2021). Heparin-mediated dimerization of follistatin. Exp. Biol. Med..

[50] Poli M., Anower-E-Khuda F., Asperti M., Ruzzenenti P., Gryzik M., Denardo A. (2019). Hepatic heparan sulfate is a master regulator of hepcidin expression and iron homeostasis in human hepatocytes and mice. J. Biol. Chem..

[51] Nemeth E., Valore E.V., Territo M., Schiller G., Lichtenstein A., Ganz T. (2003). Hepcidin, a putative mediator of anemia of inflammation, is a type II acute-phase protein. Blood.

[52] Sanz-García C., Fernández-Iglesias A., Gracia-Sancho J., Arráez-Aybar L.A., Nevzorova Y.A., Cubero F.J. (2021). The space of disse: the liver hub in health and disease. Livers.

[53] Osmond R.I.W., Kett W.C., Skett S.E., Coombe D.R. (2002). Protein–heparin interactions measured by BIAcore 2000 are affected by the method of heparin immobilization. Anal. Biochem..

[54] Abramson J., Adler J., Dunger J., Evans R., Green T., Pritzel A. (2024). Accurate structure prediction of biomolecular interactions with AlphaFold 3. Nature.

[55] Yan Y., Tao H., He J., Huang S.-Y. (2020). The HDOCK server for integrated protein–protein docking. Nat. Protoc..

[56] Olsson M.H.M., Søndergaard C.R., Rostkowski M., Jensen J.H. (2011). PROPKA3: consistent treatment of internal and surface residues in empirical pKa predictions. J. Chem. Theor. Comput..

[57] Jorgensen W.L., Chandrasekhar J., Madura J.D., Impey R.W., Klein M.L. (1983). Comparison of simple potential functions for simulating liquid water. J. Chem. Phys..

[58] Case D.A., Aktulga H.M., Belfon K., Ben-Shalom I.Y., Berryman J.T., Brozell S.R. (2022).

[59] Roe D.R., Cheatham T.E.I. (2013). PTRAJ and CPPTRAJ: software for processing and analysis of molecular dynamics trajectory data. J. Chem. Theor. Comput..

[60] Crean R.M., Pudney C.R., Cole D.K., van der Kamp M.W. (2022). Reliable in silico ranking of engineered therapeutic TCR binding affinities with MMPB/GBSA. J. Chem. Inf. Model..

[61] Schuck P. (2000). Size-distribution analysis of macromolecules by sedimentation velocity ultracentrifugation and lamm equation modeling. Biophys. J..

[62] Brautigam C.A. (2015). Calculations and publication-quality illustrations for analytical ultracentrifugation data. Methods Enzymol..

