# Supplementary material for: Erythroferrone antagonism of BMPs is governed by a composite heparin-binding motif
Source: J Biol Chem. 2026 Jun 12;302(8):113250. doi: 10.1016/j.jbc.2026.113250 (PMC13351144; doi:10.1016/j.jbc.2026.113250)
Supplement: Supplementary Figures [file mmc1.docx]

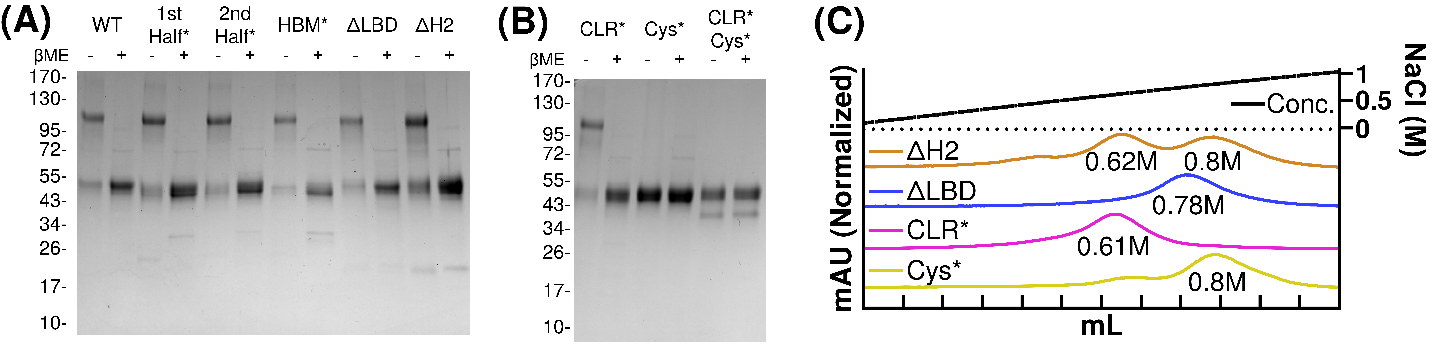


**Supplementary Figure 1**. – *Heparin affinity of purified ERFE constructs is driven by the collagen-like repeat.*

*A,B* 2 μg of WT and mutant ERFE were evaluated via SDS-PAGE with a 5-20% polyacrylamide gradient (Bio-Rad) under reducing and nonreducing conditions before being stained with Coomassie blue. *C* Heparin affinity chromatography analysis of additional ERFE mutants, eluted at a 0 to 1 M NaCl gradient. The salt concentration in molar is listed under the midpoint of each peak.


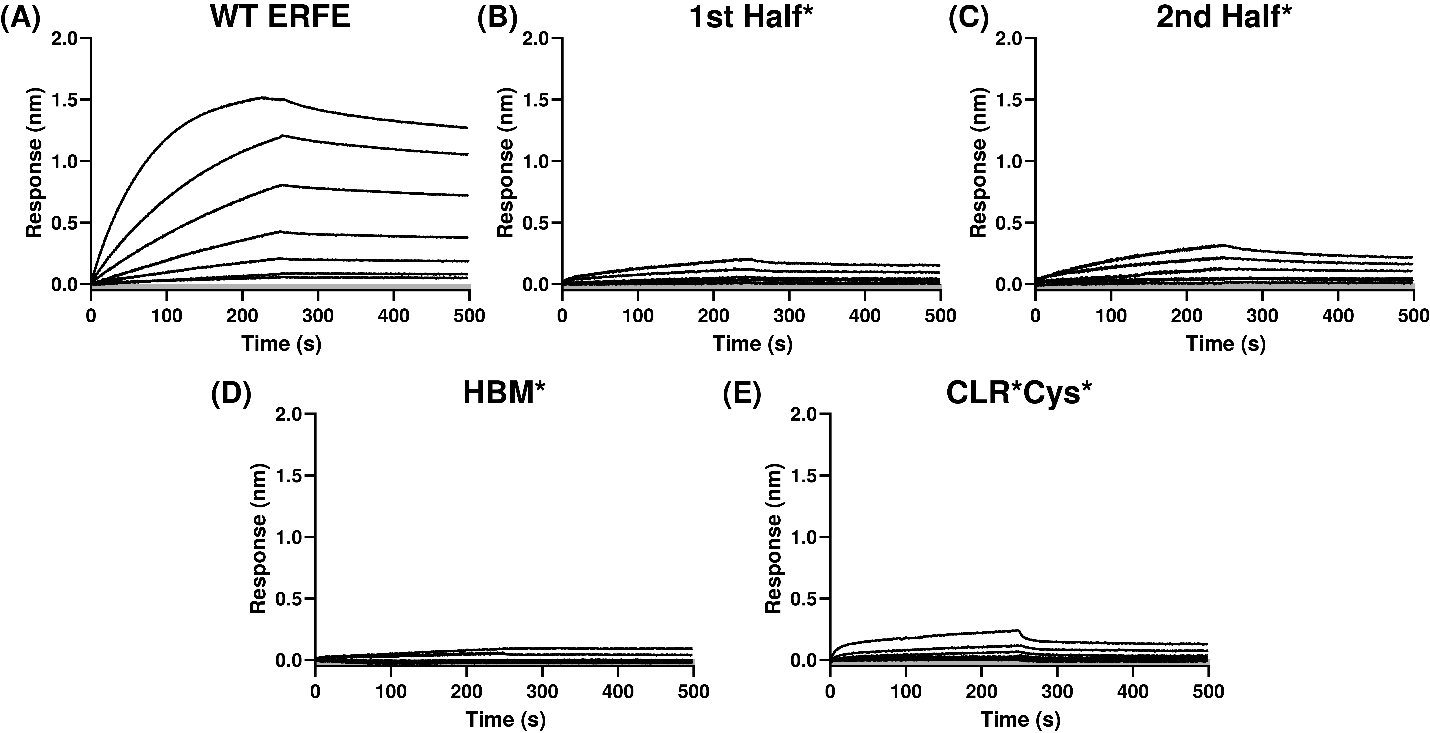


**Supplementary Figure 2.** – *Mutation of ERFE HBM dramatically decreases affinity to heparin-coupled BLI tips.*

BLI analysis of *A* WT ERFE, *B* 1st Half*, *C* 2nd Half*, *D* HBM*, and *E* CLR*Cys* binding to SA-Biotin-Heparin tips at 250 mM NaCl. Each sample was serially diluted 1:2 from 240 nM to 3.75 nM. Curves are representative of n=3 independent experiments.


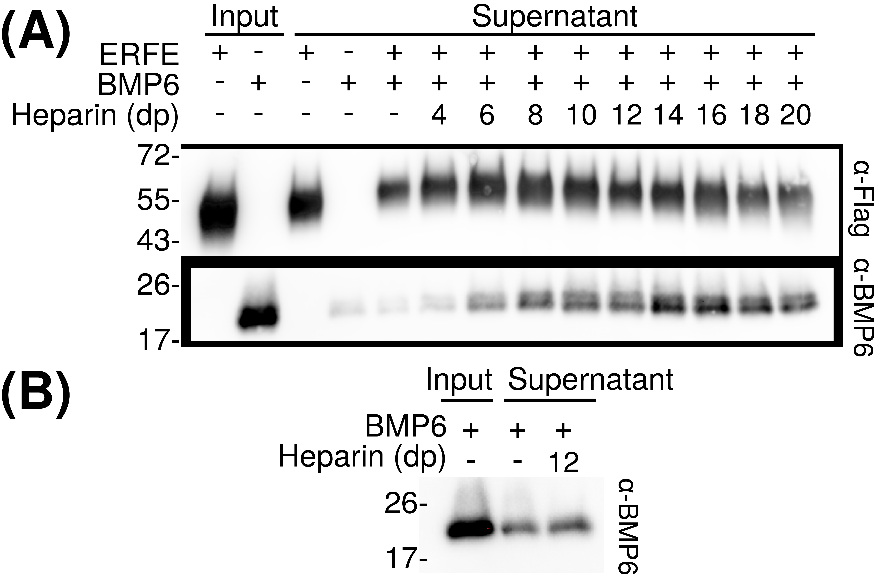


**Supplementary Figure 3.** – *The ERFE:BMP6 complex is soluble after addition of dp10 or higher chemically-defined heparin.*

*A* ERFE was complexed with BMP6 in the presence or absence of various dp lengths of chemically-defined heparin, at a 12:4:1 heparin:ERFE monomer:BMP6 molar ratio, centrifugated to remove aggregate, and the supernatant analyzed via western blot, probing for the N-terminal flag tag on ERFE or BMP6, as indicated. The western blot shown is representative of n=3 independent experiments. *B* Exogenous chemically-defined heparin added at a 12:1 heparin:BMP6 ratio did not recover BMP6 solubility.


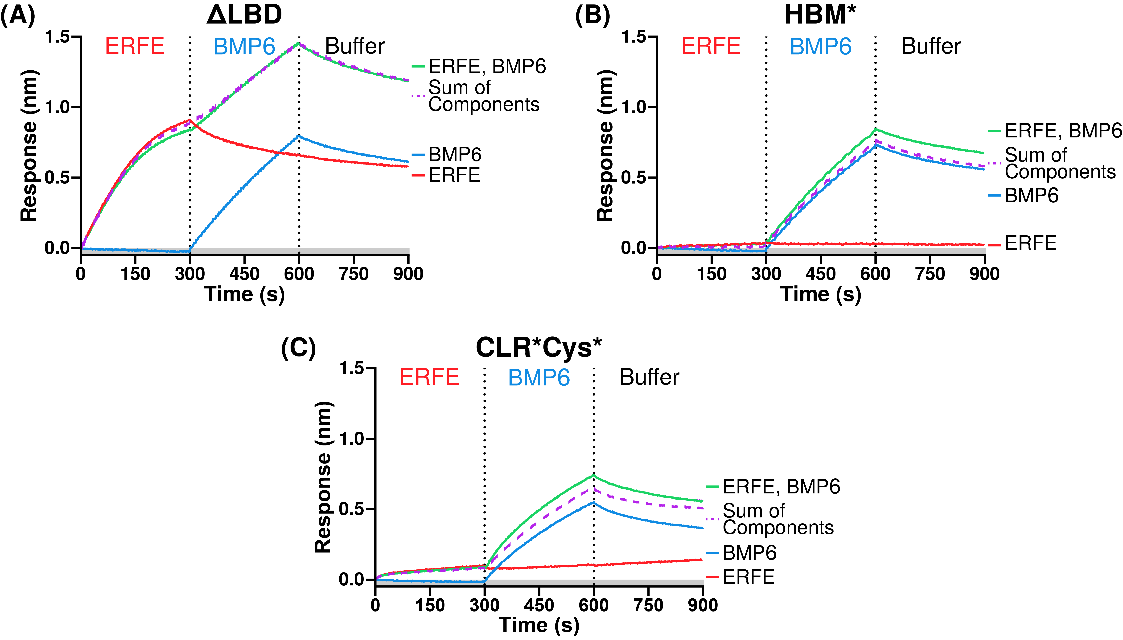


**Supplementary Figure 4.** – *Mutant ERFE is unable to synergistically complex with BMP6 after it binds heparin.*

*A* SA-biotin heparin BLI tips were bound to ERFE (red) or BMP6 (blue) and summed together to create a theoretical curve (purple) that would represent independent binding. This was compared to sequential binding of BMP6 and then ΔLBD ERFE (green), *B* HBM* ERFE, or *C* CLR*Cys* ERFE. All BLI data shown are representative of n=3 independent experiments.


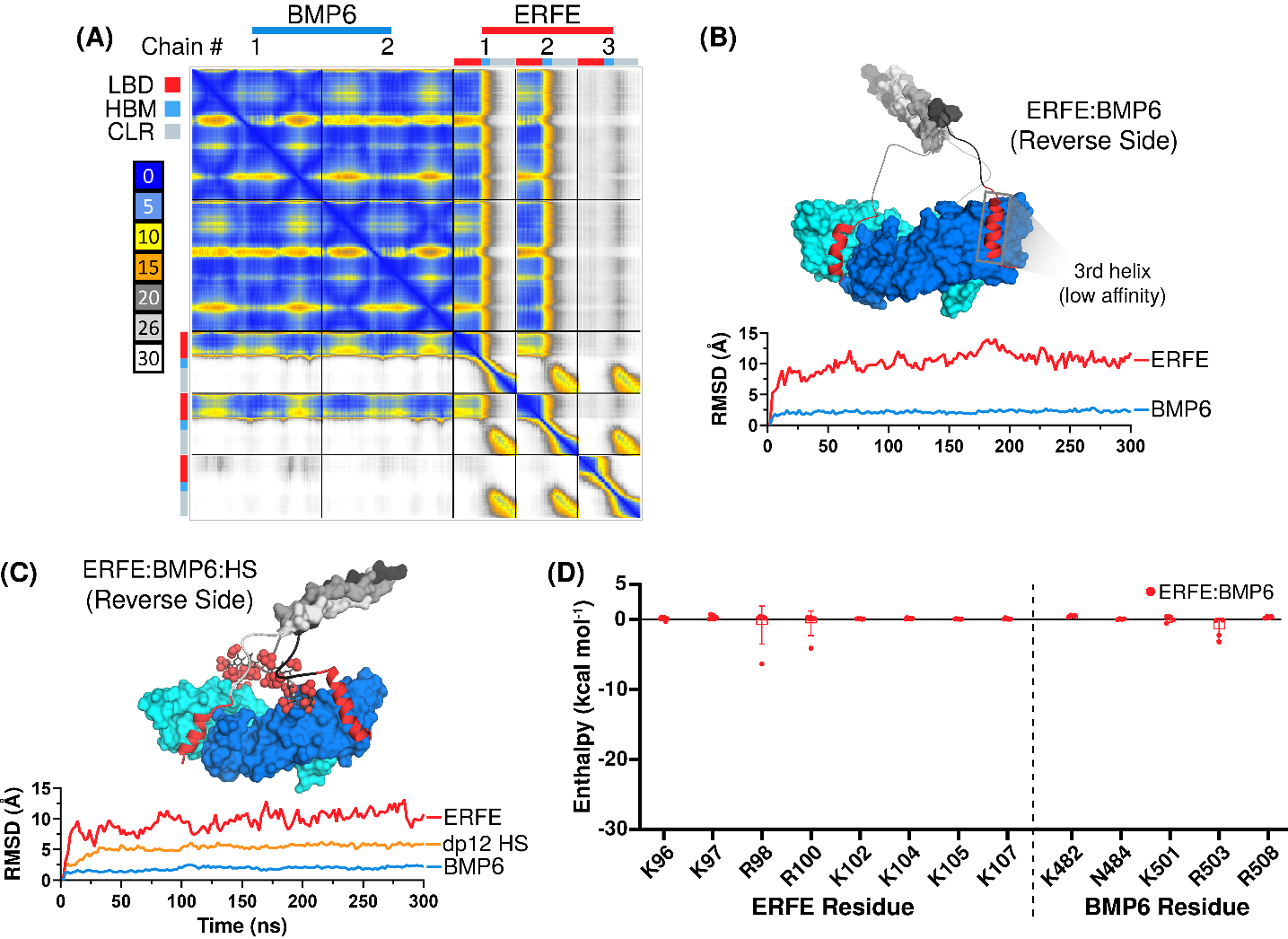


**Supplementary Figure 5.** – *Molecular dynamics simulations reach a stable equilibrium after 300 ns.*

*A* Predicted aligned error (PAE) plot of the AlphaFold3-generated model used in Fig. 3, with key regions of ERFE highlighted. *B,C* RMSD graphs from the starting position are shown for the simulations in Figs. 3 *C,D*. Note that the reverse side of the ligand is shown, and the unpaired 3rd helix is highlighted. In all graphs, ERFE is red, dp12 HS is orange, and BMP6 is blue. HS is represented using sticks with red spheres to highlight the sulfate groups. *D* MMGBSA per-residue decomposition of the enthalpy contribution of HBM residues to the stability of the ERFE:BMP6 complex without HS.


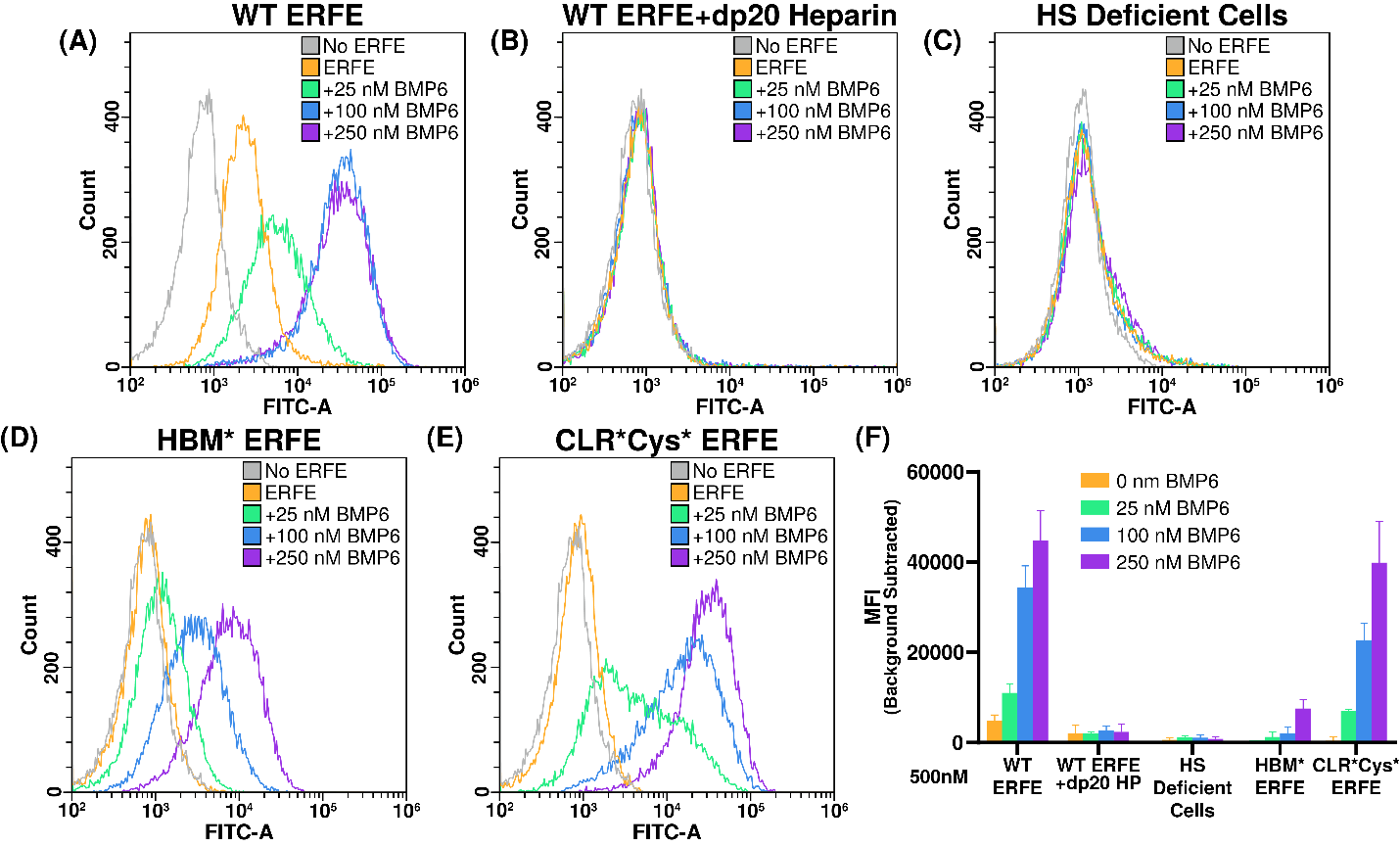


**Supplementary Figure 6**. – *Higher concentrations of ERFE cannot rescue deficiencies in HS- and ligand-dependent cell surface binding.*

*A* CHO-K1 cells were gently trypsinized and incubated with 500 nM ERFE and the listed concentration of BMP6, before probing for ERFE via anti-flag immunodetection and analysis via fluorescence detection flow cytometry. This was repeated with *B* the addition of 20:1 dp20 heparin:ERFE, *C* HS N-sulfotransferase-deficient CHO-K1 cells which lacked all HS sulfation, *D* HBM* ERFE, and *E* secondary structure-disrupting CLR*Cys* ERFE. All histograms are representative of n=3 biological replicates. *F* Quantification of MFI of n=3 replicates on a linear scale where error bars represent the S.E.M.
